# Adapting the definition of multimorbidity – development of a locality-based consensus for selecting included Long Term Conditions

**DOI:** 10.1186/s12875-021-01477-x

**Published:** 2021-06-23

**Authors:** Nasrin Hafezparast, Ellie Bragan Turner, Rupert Dunbar-Rees, Alice Vodden, Hiten Dodhia, Brian Reynolds, Barbara Reichwein, Mark Ashworth

**Affiliations:** 1 Outcomes Based Healthcare, 11-13 Cavendish Square, Marylebone, London, W1G 0AN UK; 2 Public Health Directorate, Lambeth Civic Centre, London Borough of Lambeth, 5th Floor, 2 Brixton Hill, London, SW2 1RW UK; 3 NHS South East London CCG – Lambeth Team, Civic Centre, London Borough of Lambeth, 6 Brixton Hill, London, SW2 1EG UK; 4 Guy’s and St Thomas’ Charity, 9 King’s Head Yard, London, SE1 1NA UK; 5 School of Population Health and Environmental Sciences, King’s College London, Guy’s Campus, Addison House, London, SE1 1UL UK

## Abstract

**Background:**

Defining multimorbidity has proved elusive in spite of attempts to standardise definitions. For national studies, a broad definition is required to capture national diversity. For locally based studies, the definition may need to reflect demographic and morbidity patterns. We aimed to define multimorbidity for an inner city, multi-ethnic, deprived, young age community typical of many large cities.

**Methods:**

We used a scoping literature review to identify the international literature, standards and guidelines on Long Term Condition (LTC) definitions for inclusion in our multimorbidity definition. Consensus was categorised into high, medium or low consensus, depending on the number of literature sources citing each LTC. Findings were presented to a workshop consisting of local health service stakeholders who were asked to select LTCs for inclusion in a second stage review. In the second stage, each LTC was tested against seven evaluation domains: prevalence, impact, preventability, treatment burden, progression to multiple LTCs, impact on younger people, data quality. These domains were used to create 12 target criteria. LTC rankings according to consensus group and target criteria scores were presented to a second workshop for a final decision about LTC inclusion.

**Results:**

The literature review identified 18 literature sources citing 86 LTCs: 11 were excluded because they were LTC clusters. The remainder were allocated into consensus groupings: 13 LTCs were ‘high consensus’ (cited by ≥ 11 sources); 15 were ‘medium consensus’ (cited by 5–10 sources); 47 were ‘low consensus’ (cited by < 5 sources). The first workshop excluded 31 LTCs. The remaining 44 LTCs consisted of: 13 high consensus LTCs, all with high target score (score 6–12); 15 medium consensus LTCs, 11 with high target scores; 16 low consensus LTCs, 6 with high target scores.

The final workshop selected the 12 high consensus conditions, 12 medium consensus LTCs (10 with high target scores) and 8 low consensus LTCs (3 with high target scores), producing a final selection of 32 LTCs.

**Conclusions:**

Redefining multimorbidity for an urban context ensures local relevance but may diminish national generalisability. We describe a detailed LTC selection process which should be generalisable to other contexts, both local and national.

## Background

Multimorbidity is the subject of considerable research interest because of increasing population prevalence, high concentration in elderly populations, the demands it places on traditional structures of primary care and increased secondary care utilisation [1]. People with multimorbidity have increasingly highlighted the ‘endless struggle’ of trying to live with multimorbidity, juggling the demands of healthcare access, the burden of polypharmacy and continual monitoring [2]. Reported patient experience is diminished in those with multimorbidity leading to calls for more patient-centred and holistic healthcare provision [3].

The definition of multimorbidity has proved elusive. Pioneering research in Scotland was based on a definition which included two or more of 40 Long Term Conditions (LTCs) [4]. More recently, a UK-wide study based on CPRD data adapted the Scottish definition, reducing the number of included LTCs to 36 [5]. Nevertheless, several included LTCs, while fulfilling the typical criteria of a LTC, most commonly appear as relatively minor LTCs, such as chronic sinusitis, migraine and constipation. In contrast, other LTCs may be severely disabling, such as Parkinson’s Disease, recent cancer, epilepsy and stroke. In general, the broader the definition of multimorbidity, the less severe the included LTCs. One study from Spain included 146 LTCs, many typically less severe LTCs such as lipid disorders, acne or varicose veins [6].

In defining which LTCs should be included within a definition of multimorbidity, a set of criteria are required. The O’Halloran (2004) criteria for inclusion are that the LTC should (i) have a duration of 6 months or more; (ii) have a pattern of long term recurrence or deterioration; (iii) have a poor long term prognosis and (iv) are associated with quality of life impairment [7]. Updating these criteria, Barnett et al. (2012) included LTCs which were: *‘… likely to be chronic (defined as having significant impact over at least the most recent year) and with significant impact on patients in terms of need for chronic treatment, reduced function, reduced quality of life, and risk of future morbidity and mortality’* [4]. Comparing these sources alongside NICE and Department of Health definitions for a LTC, there was a consensus for the definition of a LTC for this study to be:

*‘Health conditions for which there is currently no cure, but which can be managed with drugs and other treatments. A LTC is one that lasts a year or longer, and impacts on a person’s life’.*

Whilst reviewing existing definitions for multimorbidity, most defined this as being ‘two or more long-term conditions which are either physical or mental health conditions’. In a challenge to these definitions of multimorbidity, the Academy of Medical Sciences emphasised the importance of including chronic infections such as hepatitis B, hepatitis C and HIV infection [8]. Hepatitis B and hepatitis C had been included in both the Barnett et al. and Cassell et al. studies, although subsumed under ‘viral hepatitis’ or ‘chronic liver disease’; neither had included HIV infection [4, 5]. On this basis, the consensus definition for multimorbidity for this study was that of the Academy of Medical Sciences:

The presence of two or more LTCs each of which is either:

- A physical non-communicable disease of long duration, such as a cardiovascular disease or cancer.
- A mental health condition of long duration, such as a mood disorder or dementia.
- An infectious disease of long duration, such as HIV or hepatitis C.

A further element in the selection of LTCs included in the definition of multimorbidity is that classification should be, ‘*driven by the purpose of measurement, but will inevitably be at least partly subjective and partly pragmatic…all multimorbidity measures are therefore contestable, but the choices made should be as explicit as possible*’ [4]. A key distinction is whether multimorbidity is defined for research or clinical purposes. For national studies, a broader definition would be preferable in order to encompass national diversity. For local studies, the broader definition of multimorbidity may insufficiently reflect local priorities such as demography, epidemiology, health care utilisation and patient experience.

For this study, we aimed to devise a definition of multimorbidity reflecting the pattern of LTCs in an inner-city, high density, deprived, multi-ethnic community, and to develop a methodology for defining multimorbidity in local populations which might be more generally applicable. The wider purpose of this study was to understand the progression patterns towards multimorbidity within a local community, including common sequences of LTC diagnoses in an urban population with multimorbidity.

## Methods

Our aim was to create a taxonomy of multimorbidity which reflected the characteristics of an inner London locality, the boroughs of Lambeth and Southwark. The combined population of these boroughs is 640,000; 44% are of non-white ethnicities (25% Black; 9% Asian) although the proportion of white ethnicity increases in the over 75’s [9, 10]. The combined population is relatively young compared with the overall UK population: 8% aged 65 years or over compared with a 18% for England as a whole. Both boroughs are characterised by high levels of social deprivation: Southwark is the 41^st^ most deprived local authority; Lambeth 44^th^ most deprived, out of 326), although mixed with small areas of considerable prosperity.

In this study, we describe the process of defining which LTCs to include in our definition of multimorbidity and the final selection of LTCs. We confined our definition to adults aged 18 years and over.

The process of defining the final selection of LTCs was a multi-stage exercise, punctuated by two consensus-building workshops with local public health and academic experts, health service commissioners, clinicians, third sector and community representation from HealthWatch. The first part was a thorough scoping review of national and international literature, standards and guidelines on LTC definitions and LTCs considered to be an LTC within these definitions. Seventeen commonly cited sources were included (see Table 1), as well as a list of LTCs in an earlier piece of work on multimorbidity in Southwark and Lambeth [1] (eighteen sources in total). These sources were a mixture of primarily UK sources, but supplemented with a small number of high-profile international sources.

**Table 1:** Sources reviewed in analysis of national and international consensus on the definition of LTCs

**Guy’s and St Thomas’ Charity** Report on multiple LTCs [11]	**Quality and Outcomes Framework** LTC registers [12]	**NHS Outcomes Framework** Chronic Ambulatory Care Sensitive LTCs indicator [13]
**GP Patient Survey** Self-reported LTCs (survey) [14]	**Ipsos MORI** Self-reported LTCs (research study) [15]	**Department of Health** Compendium of Information on LTCs [16]
**North West London Whole Systems Integrated Care (WSIC)** LTC segment definition [17]	**Managing Long-term Conditions and Chronic Illness in Primary Care** LTC management [18]	**NHS Scotland** LTC report [19]
**Australian Institute of Health and Welfare** Chronic disease report [20]	**British Columbia Ministry of Health** Segment definition (Living with Illness and Chronic Conditions) [21]	**World Health Organisation** Report on non-communicable diseases [22]
**Epidemiology of multimorbidity. Barnett et al** Paper on LTCs/multimorbidity [4]	**From chronic conditions to relevance in multimorbidity. N’Goran et al** Paper on LTCs/multimorbidity [23]	**Health Foundation** Briefing on LTCs/multimorbidity [1]
**Centers for Medicare and Medicaid Services** Definition of chronic conditions [24]	**Centers for Disease Control and Prevention** Definition of chronic conditions [25]	**NHS England, RightCare** LTC data packs [26]

Any LTC mentioned in these definitions was included in an initial comprehensive list of all LTCs available for consideration. Synonyms for the same LTCs named differently across different sources were standardised. A count of sources that mentioned each LTC was then calculated for each LTC. This score (from eighteen possible sources) was treated as a consensus literature score (i.e. a higher score represented a LTC that was more commonly included across LTC definition sources).

We stratified the scores creating three distinct groups of LTCs based on pragmatic groupings of the number of literature sources. LTCs with high consensus (included by 11 or more sources), with medium consensus (5–10 sources), and low consensus (1–4 sources). Certain risk factors were considered as LTCs by some sources and risk factors by others. Where this was the case, they were included in the list of LTCs.

An initial workshop was held with local stakeholders (primary care, public health, local care networks) to review this full list of LTCs stratified by consensus grouping. It was agreed that low consensus LTCs should be removed from further evaluation at this stage, unless there was a local reason to include.

The first workshop was designed to select a list of included LTCs for further consideration. During this initial workshop it was agreed to use seven evaluation domains to analyse each of these LTC’s more extensively. These domains were designed to ensure locally important LTCs were identified, and that the LTCs matched the consensus definition of an LTC described in the Background section:

1. **Prevalence**: a review of national prevalence data ensured that no high prevalence LTCs were missing from the definition. Sources of prevalence data included the Quality and Outcomes Framework (2017/18) [27], reports from charities and national bodies, academic papers and Global Burden of Disease (GBD) estimates [28]. We cross-referenced these sources against the chronological map of 308 LTCs published by Kuan et al. [29]. All estimates were standardised to the UK population size in 2017 for comparison. LTCs with greater than 1% prevalence were included on this basis.
2. **Impact**: Assessment of impact involved three separate criteria. Firstly, the impact of LTCs was assessed on whether or not LTCs followed a progressive natural course, with increasing severity over time. Secondly, the total population burden of years of life lost (YLL) and, thirdly, years of life spent in disability (YLD) were also analysed to understand the relative burdens of mortality and morbidity associated with each LTC. The top 20 LTCs ranked by burden of YLL, and YLD were each selected as ‘high impact burden’ LTCs for each criterion separately. This was based on GBD data. Causes from the GBD study were mapped as closely as possible to LTCs in the list. Additional factors were considered (e.g. whether LTCs followed a stable course, or whether they were relapsing/remitting, or punctuated by acute episodes, and whether it is possible to be asymptomatic with the LTC), however, these factors were found to be too ambiguous to be used as a decision-making factor. They were maintained in the analysis to provide additional context.
3. **Preventability and modifiability**: these are important factors to ensure that primary or secondary prevention action is possible to either reduce the prevalence of the LTC or slow progression of the LTC. A clinical literature review was conducted to identify LTCs with evidence of risk factors playing a role in preventing or delaying its onset. Specific focus was given to the ‘Vital 5 risk factors’, identified locally as of particular importance in the initiation and development of LTCs [30]. LTCs for which an intervention could result in complete resolution were also identified. Both were considered as binary yes/no criteria.
4. **Treatment burden**: treatment burden was assessed both in terms of admitted patient care burden, and medication burden. Outpatient appointment burden was also considered, but the quality of diagnoses recorded in outpatient care data did not provide a sufficiently complete view. Admitted patient care burden was based on Hospital Episode Statistics data, and was assessed as the number of admissions with a primary diagnosis of the relevant LTC [31]. The top 20 LTCs by admitted patient burden were identified as high-care-burden LTCs. Medication burden was difficult to classify, due to changes in relation to severity and stage. It was defined as the number of discrete first-line medications for the purposes of comparing between LTCs [32]. Those with two or more first-line medications were considered to be LTCs with high medication burden.
5. **Progression to multiple LTCs**: as the focus of the study was the journey to multimorbidity, multimorbidity itself was one of the evaluation criteria. A literature review provided estimates of the proportion of people with each LTC to be comorbid with at least one other LTC. Whilst sources may have included different LTCs in their definition of multimorbidity, the aim was to differentiate between LTCs commonly coinciding with other LTCs, from LTCs that are more likely to exist as a sole LTC. A binary cut-off of 50% comorbid was therefore taken as an indication of LTCs that are heavily associated with multimorbidity.
6. **Relative impact on younger people**: another study-specific criterion reflecting local population demographic characteristics was to avoid omitting LTCs likely to occur as the first in a sequence of LTCs towards multimorbidity. Accurate measurement of the sequencing of LTCs usually requires linked local healthcare datasets, which was not available at this stage of the study, therefore we identified LTCs that are more likely to affect younger people, making them more likely to be the first in a sequence of LTCs. LTCs that were likely to impact people aged under 50, were identified through average age at onset data [29] as well as LTCs with a high YLD burden in the 15-49 age group (based on GBD data, defined as the top 20 LTCs by YLD burden in this age group).
7. **Data quality**: a final criterion was whether or not each LTC could be identified sufficiently accurately in primary and/or secondary care datasets. Whereas all other criteria were criteria of inclusion (i.e. accumulating reinforcement across criteria for inclusion of the LTC within the definition of LTCs), data quality was a potential exclusion criterion, given that it would prevent analysis from being undertaken. Each LTC was assigned a ‘high’, ‘medium’ or ‘low’ level of data integrity, based on combined knowledge and experience of these datasets, with three further, more tangible criteria: firstly, whether a LTC is included in the Quality and Outcomes Framework (QOF), in which case it is likely to be well coded in primary care; secondly, whether a LTC is likely to lead to frequent admissions to hospital, in which case it is likely to be well coded in secondary care; and finally, whether a LTC requires regular prescriptions, which increases the likelihood of it being well coded in either dataset, but particularly in primary care. ‘Low’ data quality LTCs were excluded from the definition (but could be reinstated if data quality improved).

There were 12 target criteria set for these 7 evaluation domains, shown in Table 2. This enabled a total score to be generated, showing the total number of target criteria that were met, as applied to each LTC. We stratified the scores creating two distinct groups based on a pragmatic median cut-point creating two groupings: target criteria score ≥ 6 or target criteria score < 6. These ‘scores’ and groupings were used for discussion at a second workshop held with local stakeholders (primary care, public health, local care networks) to make the final decision to include or exclude LTCs for the final list of LTCs to be included in our locally determined definition of multimorbidity.

**Table 2:** Target criteria set for each evaluation domain

**Domain**	**Target Criteria**	**Inclusion Criteria**
**Prevalence** Purpose: to identify high prevalence conditions (that impact a greater number of people)	Estimated condition prevalence (UK 2017)	> = 1%
**Impact of LTC** Purpose: to identify conditions that have a greater impact on people’s lives	Progressive natural course? (yes/no)	Yes
**Impact YLL** Purpose: to identify conditions that are having a greater population-level impact in terms of years of life lost	Rank by volume of YLL (UK, 2017—Source: GBD)	Top 20
**Impact YLD** Purpose: to identify conditions that are having a greater population-level impact in terms of years lived in disability	Rank by volume of YLD (UK, 2017—Source: GBD)	Top 20
**Prevention and Modifiability** Purpose: to identify conditions that can be prevented, the onset delayed, or improved by modifying risk factors or intervention	Do risk factors play a role in preventing or delaying the onset of the condition? (yes/no)	Yes
	Can intervention result in complete resolution? (yes/no)	Yes
**Treatment Burden: Utilisation** Purpose: to identify conditions that account for a high-proportion of population-level admitted patient care	Rank by volume of hospital admissions (based on primary diagnosis) (England, 2017/18—Source: HES data, NHSD)	Top 20
**Treatment Burden: Medication** Purpose: to identify conditions that have a high treatment burden, particularly in relation to medication burden	Number of first-line, self-administered medications	> = 2
**Progression to mLTCs** Purpose: to identify conditions that are most likely to be involved in a mLTCs journey	Proportion of people with the condition who have 1 + comorbidities	> 50%
**Impact on younger people: Age at Onset** Purpose: to identify conditions that can present in younger people, as these that are more likely to be the first condition in a multimorbidity pathway	Typical age of onset of the condition	< 50 years old
**Impact on younger people: YLD in younger people (aged 15–49)** Purpose: to identify conditions that have a high population-level impact on years lived with disability, in younger people	Rank by volume of YLD, in people aged 15–49 (UK, 2017—Source: GBD)	Top 20
**Data Quality** Purpose: to identify conditions where data quality is of a sufficient level to allow for meaningful data analysis	Level of data quality: Low/Medium/High, based on three main criteria (whether a condition is included in QOF, whether regular/frequent prescriptions are required, whether hospitalisation for the condition is common), in combination with background knowledge on data quality	Medium and High

## Results

**Fig. 1:**
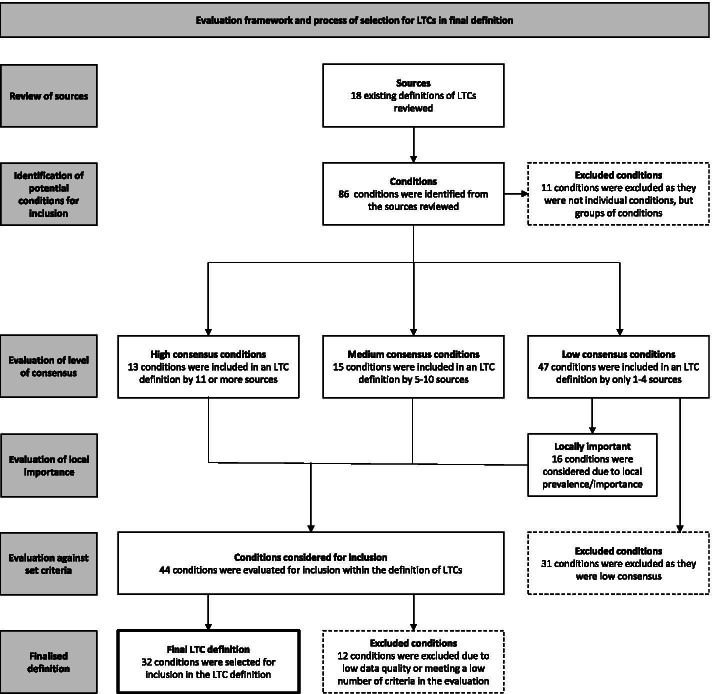
Process of evaluation of LTCs for inclusion in the definition of multimorbidity

The initial scoping literature review identified 86 LTCs. Following the evaluation methodology summarised in Fig. 1, 32 LTCs were included in the final list of LTCs for the local definition of multimorbidity.

### Excluded LTC clusters

Eleven LTCs were excluded as these were considered grouped categories of LTCs, rather than individual LTCs. These were: Circulatory Conditions, Cardiovascular Disease (CVD), Gastrointestinal Disorders, Infectious Diseases, Mental Health Conditions, Musculoskeletal Conditions / Rheumatic Disease, Neurological Conditions, Respiratory Conditions, Skin Conditions, Other Chronic Conditions, Substance Misuse. Each LTC within these broad classifications was considered separately.

### Evaluation of consensus based on literature sources

The remaining 75 LTCs were grouped into high consensus LTCs i.e. those included by 11 or more sources (*n* = 13 LTCs), medium consensus LTCs i.e. those included by 5–10 sources (*n* = 15 LTCs), and low consensus LTCs i.e. those included by 1–4 sources (*n* = 47 LTCs).

Low consensus LTCs were reviewed at the first local stakeholder workshop at which it was agreed to consider exclusion on the basis of being low consensus, except for locally important LTCs which would be included for further evaluation. Several locally prevalent and/or important LTCs were included on this basis (e.g. HIV, sickle-cell anaemia). On this basis, 31 of the 47 low consensus LTCs were excluded; the remaining 16 LTCs were included for further evaluation (Table 3). Overall, the first workshop reduced the list of included conditions from 75 to 44 LTCs.

**Table 3:** Summary of low consensus LTCs based on literature search, including those locally important considered for inclusion in the definition of multimorbidity (*n* = 47)

**Alcohol Dependence**	‘Medication Abuse’
**Anorexia or Bulimia**	Memory Disturbance
**Back Pain**	**Migraines**
Bowel Incontinence	Motor Neurone Disease (MND)
Bronchiectasis	**Obesity**
Cerebral Palsy	**Obsessive–Compulsive Disorder (OCD)**
Chronic Fatigue Syndrome (CFS)/ Myalgic Encephalomyelitis (ME)	Other Facial Pain
Chronic Sinusitis	**Other Inflammatory Polyarthropathies and Systemic Connective Tissue Disorders**
Chronic Skin Ulcer	Substance dependence
Constipation	**Personality Disorder**
Cystic Fibrosis	**Phobias**
Diverticular Disease/Diverticulitis	Polio
‘Drug Abuse’	**Post-Traumatic Stress Disorder**
Dyspepsia	Prostate Disorders
Eczema	Psoriasis
Endometriosis	Rare Long-Term Neurological LTCs
Frailty	**Sickle Cell Anaemia**
Gout	‘Tobacco Abuse’
**Headache**	Tooth Decay
High Cholesterol	Trigeminal Neuralgia
**HIV/AIDS**	Urinary Incontinence
Irritable Bowel Syndrome (IBS)	Urinary System LTCs
**Liver Disease (chronic)**	**Viral Hepatitis (B & C)**
**Lupus**	

LTCs in highlighted in bold (*n* = 16) were considered locally important and included for further evaluation

### Evaluation of consensus based on domain criteria

The remaining 44 LTCs were then considered against 12 target criteria shown in Table 2.

#### High consensus LTC target criteria scores (*n* = 13)

All high consensus LTCs were included in the final definition of LTCs (Table 4). Each met at least six of the 12 target criteria (the lowest number of criteria met in this high consensus group was seven).

**Table 4:** Summary of high, medium and low consensus LTCs considered for inclusion in the definition of multimorbidity based on target criteria

**High consensus LTCs**^**a**^	**Medium consensus LTCs**^**b**^	**Low consensus LTCs**^**c**^
**Asthma**	**Anxiety Disorders**	**Alcohol Dependence**
**Atrial Fibrillation**	Blindness/Severe Visual Impairment	Anorexia or Bulimia
**Cancer**	**Chronic Pain**	Back Pain
**Chronic Kidney Disease**	**Cognitive and Learning Disabilities**	Headache
**COPD**	Deafness/Severe Hearing Impairment	**HIV/AIDS**
**Coronary Heart Disease**	**Inflammatory Bowel Disease**	**Liver Disease**
**Dementia**	**Multiple Sclerosis**	**Lupus**
**Depression**	**Osteoarthritis**	Migraines
**Diabetes**	**Osteoporosis**	Obsessive–Compulsive Disorder (OCD)
**Epilepsy**	**Parkinson’s Disease**	Other Inflammatory Polyarthropathies & Systemic Connective Tissue Disorders
**Heart Failure**	**Peripheral Arterial/Vascular Disease**	Personality Disorder
**Hypertension**	**Rheumatoid Arthritis**	Phobias
**Stroke**	**Serious Mental Illness**	Post-Traumatic Stress Disorder
	Thyroid Problems	**Sickle Cell Anaemia**
	**Transient Ischaemic Attack**	**Viral Hepatitis (B & C)**

^a^All high consensus LTCs (*n* = 13) met the target criteria score and included for further evaluation

^b^The medium consensus LTCs highlighted in bold (*n* = 12) met the target criteria score and included for further evaluation

^c^The low consensus LTCs highlighted in bold (*n* = 7) were considered locally important and included for further evaluation

#### Medium consensus LTC target criteria scores (*n* = 15)

Eleven medium consensus LTCs had a target criteria score ≥ 6 (Table 4). All were included, except for thyroid problems. Emphasis was placed on the impact that LTCs have on people’s lives, and it was considered that the relative impact of thyroid problems compared to other LTCs was low once treated.

Of the four remaining medium consensus LTCs with a target criteria score < 6, two were excluded on the basis of low data quality (blindness/severe visual impairment; and deafness/severe hearing loss). Two LTCs (Peripheral Arterial Disease and Transient Ischaemic Attack) met less than six criteria but were included due to a local focus on cardiovascular LTCs.

#### Low consensus LTC target criteria scores (*n* = 16)

Six low consensus LTCs had a target criteria score ≥ 6 (Table 4). Three of these were included (alcohol dependence, chronic liver disease and morbid obesity) on the basis of high local prevalence in urban communities. Two were excluded on the basis of low data quality (back pain and headache). One LTC (migraine) was excluded because it was considered that it would be included as a subset of chronic pain (one of the included medium consensus LTCs).

Ten low consensus LTCs had a target criteria score < 6. Five were excluded on the basis of low data quality (anorexia/bulimia, Obsessive Compulsive Disorder, Post Traumatic Stress Disorder, phobias and personality disorder). Other inflammatory polyarthropathies and systemic connective tissue disorders were also excluded on the basis of the low consensus and low number of criteria met. Four LTCs were included despite the low consensus and low number of criteria met, due to high local prevalence: HIV/AIDs, viral hepatis (B and C), Sickle-Cell Anaemia, and a large local at risk population (Lupus).

### Final workshop review

**Fig. 2:**
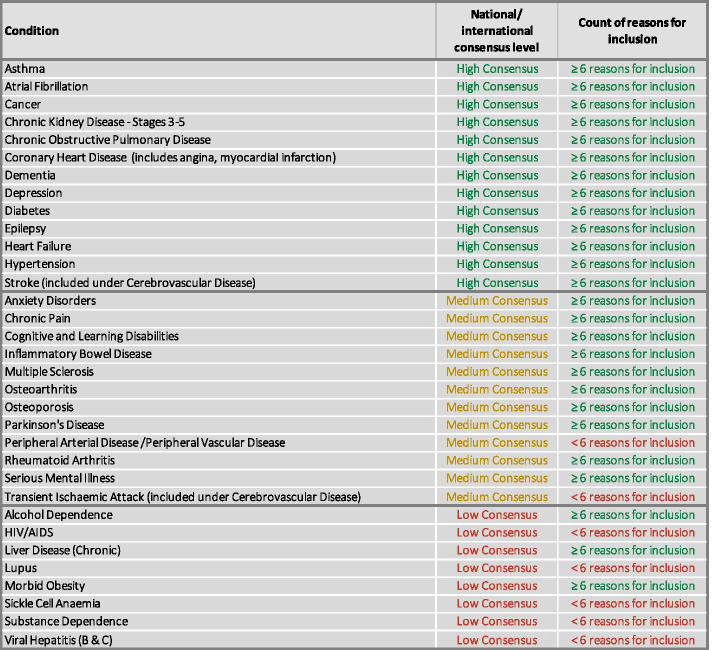
Final list of LTCs included in definition of multimorbidity

**Fig. 3:**
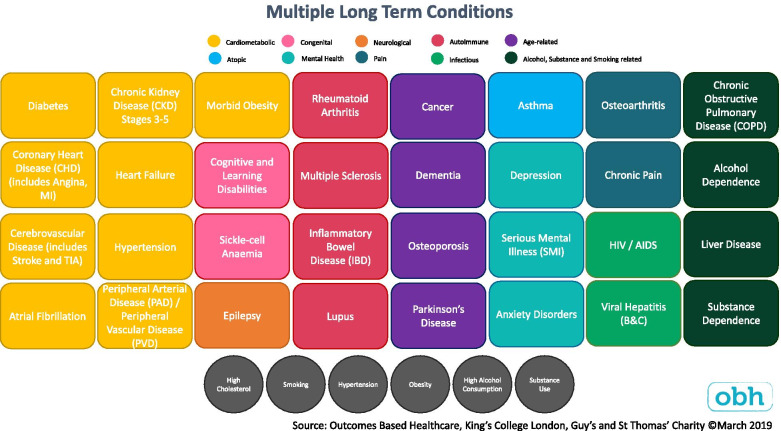
Graphic of LTCs included in definition of multimorbidity

At the end of the second workshop, the list of 32 LTCs included in the definition of multimorbidity was reviewed. Based on local importance and local prevalence, the decision to exclude substance dependence was reviewed; it had been removed based on the evaluation of literature sources (Table 4) and a decision was taken to reinstate substance dependence as an LTC. It was also considered that Transient Ischaemic Attack (TIA) should be included with Stroke under Cerebrovascular Disease and not as a separate entity given the similarity in pathophysiology between the conditions, and in line with other authors [4, 5]. Consensus was checked at both workshops, was inclusive such that disagreement resulted in inclusion of the LTC until further stages of the consensus process; there was unanimous agreement on the final list of LTCs. The final list of included LTCs is described in Fig. 2 and summarised in Fig. 3.

## Discussion

Multimorbidity as a concept lacks consensus about which LTCs to include. National definitions of multimorbidity may have less relevance when applied to populations within localities, particularly where those localities differ substantially in demographic or morbidity characteristics from overall national characteristics.

We present a process for selecting LTCs to be included within a locality based consensual definition of multimorbidity. The process described consisted of five steps: literature review, selection of LTCs based on literature sources and ranked according to literature source consensus, first consensus workshop, application of criteria agreed at first consensus workshop, second consensus workshop to finalise selection of LTCs.

The essential components of this process were ‘consensus’ and ‘locality’. This approach to seeking consensus on a definition of MLTCs was seen as an essential enabler for any subsequent analysis and intervention to slow progression from one to many LTCs [11]. Without a common definition of the scope of MLTCs, designing appropriate interventions risks becoming an unfocussed exercise, and evaluation of any interventions becomes substantially more challenging. This work to define multimorbidity should be seen as a foundation to a wider programme of work, rather than an end in itself.

### Comparison with the literature

We were unable to find other examples in the literature describing a stakeholder consensus approach following defined steps which could be replicated in other settings for the creation of an agreed set of LTCs constituting a definition of multimorbidity. Other reports have highlighted the importance of using research definitions based on the outcomes of interest [33]. Thus, for example, some definitions may focus on patient quality of life, others on healthcare utilisation, or on varying balances between mental health and physical health LTCs. In recent developments, some have developed a more holistic approach including risk factors [33]. The boundary between risk factor and LTC becomes blurred with LTCs such as hypertension which may be considered as both, and artificial thresholds for disease which applied to our inclusion of morbid obesity (as opposed to moderate obesity), alcohol dependence (as opposed to consumption in excess of 14U/week) and substance dependence (as opposed to substance use). Whether these distinctions can be accurately captured within existing primary care datasets remains to be seen.

In a Delphi consensus exercise, 229 European ‘medical experts’ attempted to define multimorbidity [34]. Although consensus methods were used, the definition of multimorbidity had to be generalisable, applying to widely varying primary care systems across all European countries and with outcomes of interest ranging from research to direct patient care and resource allocation. The diversity of population demographics, primary healthcare systems and outcomes of interest across a whole continent may dilute the potential impact of a more universal approach to defining multimorbidity.

Our detailed process for selecting LTCs for inclusion in a locally based definition of multimorbidity resulted in an overall list of LTCs similar to the list produced initially by Barnett et al. [4] and more recently revised by Cassell et al. [5]. Of the 32 LTCs included in our selection, morbid obesity, osteoarthritis, HIV and sickle cell disease were unique to our selection; lupus as an individual LTC was also unique to our selection, although subsumed into a single broader category by Cassell et al. which also included rheumatoid arthritis and systemic connective tissue disorders. Our selection process excluded the following conditions which were included by Cassell et al.: anorexia, blindness, bronchiectasis, chronic sinusitis, constipation, diverticular disease, hearing loss, irritable bowel syndrome, migraine, prostate disorders, psoriasis/eczema, thyroid disease. Some, but not all, of these conditions excluded in our selection may be characterised by less severe ‘impact’ and ‘treatment burden’ (Target Criteria domains 2&4).

### Strengths and limitations

In constructing a locality based definition of multimorbidity, it is likely that we have sacrificed generalisability for local applicability. For example, our definition is unlikely to apply to less deprived areas with more mono-ethnic or older population structures. Nevertheless, many inner-city areas are characterised by younger, multi-ethnic populations for which our definition may be well adapted. Similarly, our emphasis on local relevance meant that the literature sources included in the consensus exercise were predominantly UK based.

The use of local consensus to develop a working definition of multimorbidity is likely to contribute to eventual use of this definition for local prevention of multimorbidity or reduction of its consequences. However, our focus has excluded children and teenagers which may be relevant for LTCs or risk factors involving younger populations [29]. Similarly, the broader focus on managing multimorbidity meant that we excluded end-of-life care. Recent work has emphasised the importance of weighting LTCs according to the outcome of interest [35] and to ensure generalisability, we have not attempted to differentiate between stable LTCs (such as early COPD, CKD, Heart Failure) or the same LTCs when end-stage. Similarly, practical difficulties with interpreting primary care data meant that we were unable to differentiate between previous cancer with no recurrence (possibly cured) or cancer with secondary spread. Our definition did not include measures of functional impairment nor the perceived burden for patients, again because of limitations of primary care data availability. A further limitation arose because not all seven ‘evaluation criteria’ could be objectively assessed and some, like ‘impact of LTC’ was largely subjective in its application. Our study was limited to selecting LTCs for inclusion in a definition of multimorbidity and did not consider which categories of each LTC should be included (where categories exist) nor which Read or SNOMED codes should be used to define each included LTC which will be the subject of further work. Similarly, although each individual condition was considered on the basis of ‘impact of LTC’, multimorbidity itself, as a combination of two or more LTCs, may have ‘impact’ in terms of functional incapacity, debility or mortality which is not merely the sum of impacts of individual conditions.

### Implications for practice

Re-defining multimorbidity in terms of a locality consensus has implications for our understanding of the nature of LTCs themselves, particularly in terms of how they relate to each other within an urban context. Importantly, this allows us to ask questions about the sequencing of specific LTCs. For example, are there a number of potential ‘gateway LTCs’ (e.g. depression, hypertension) [36] which, if managed appropriately, could delay or prevent progression to other LTCs? Or are such high incidence, young age of onset, LTCs merely a sequencing artefact of being the first to occur, and bearing no relation to the development of subsequent LTCs?

Ultimately it is essential to appreciate that any data analysis subsequent to this kind of exercise should not be a static exercise. A dynamic, longitudinal perspective will be essential to understand population-level ‘flow rates’ between these health states and multimorbidity, in order to evaluate the effectiveness of any exercise or intervention designed to slow progression. This requires relatively sophisticated datasets, and data analysis, which could limit the broader local application of this work- at least in the short term.

## Data Availability

The datasets used and/or analysed during the current study are available from the corresponding author on reasonable request.
